# Real-World Effects of Single-Inhaler Fluticasone Furoate/Umeclidinium/Vilanterol in Chinese Patients with Asthma

**DOI:** 10.3390/jcm15207990

**Published:** 2026-10-15

**Authors:** Anqi Li, Zhouxian Pan, Juan Liu, Lun Li, Xin Li, Zhirong Du, Jia Yin

**Affiliations:** Department of Allergy, Beijing Key Laboratory of Precision Medicine for Diagnosis and Treatment on Allergic Diseases, Chinese Academy of Medical Sciences & Peking Union Medical College Hospital, Beijing 100730, China

**Keywords:** asthma, fluticasone furoate/umeclidinium/vilanterol, triple inhaled therapy, lung function, real-world study, allergen immunotherapy

## Abstract

**Background/Objectives:** Asthma is a heterogeneous chronic inflammatory airway disease. In China, achieving rapid asthma control remains a major challenge, and a substantial proportion of patients remain inadequately controlled despite regular use of standard inhaled corticosteroid/long-acting β_2_-agonist (ICS/LABA) therapy. Fluticasone furoate/umeclidinium/vilanterol (FF/UMEC/VI) has demonstrated clinical efficacy in trials, but its real-world effectiveness and safety in China remain insufficiently characterized. This study evaluated FF/UMEC/VI (100/62.5/25 μg) in Chinese patients with uncontrolled asthma. **Methods:** This retrospective study included patients with asthma treated with FF/UMEC/VI at the Department of Allergy, Peking Union Medical College Hospital, between July 2021 and March 2024. The cohort included patients with no prior inhaled medication, irregular prior inhaled therapy, or regular prior inhaled therapy with inadequate control, reflecting a real-world spectrum of patients requiring treatment intensification. Assessments were performed at baseline and 1, 3, and 6 months after treatment initiation. The primary endpoint was the change in forced expiratory volume in 1 s (FEV_1_) at 1 month. Secondary outcomes included changes in FEV_1_, percent predicted FEV_1_, and FEV_1_/forced vital capacity (FEV_1_/FVC), and safety. **Results:** Thirty patients were analyzed. FEV_1_ increased significantly at 1 month and remained above baseline at 3 and 6 months. Percent predicted FEV_1_ and FEV_1_/FVC also improved significantly at all follow-up visits. Maintenance therapy was reduced in 53.3% of patients. No serious adverse events occurred; reported events were mainly local throat discomfort and increased sputum production. **Conclusions:** In real-world practice, FF/UMEC/VI was associated with improved lung function measures in Chinese patients, including previously untreated, irregularly treated, and regularly treated but uncontrolled patients, with an acceptable safety profile. Larger prospective studies are warranted to confirm its long-term effectiveness and identify appropriate patient populations in China.

## 1. Introduction

Asthma is a heterogeneous chronic inflammatory airway disease characterized by recurrent wheezing, dyspnea, chest tightness, and cough, together with variable expiratory airflow limitation. Its prevalence and disease burden have increased substantially in China over recent decades. Epidemiological data show that the overall prevalence of asthma in mainland China reached 2.20%, with a continuous upward trend reported between 1984 and 2021 [1]. From 1990 to 2021, the burden of asthma remained particularly high among children aged 0–9 years and adults aged ≥60 years [2]. Despite this growing burden, asthma remains underdiagnosed and inadequately managed among Chinese adults. Only 28.8% of patients reported a previous physician diagnosis, 23.4% had undergone pulmonary function testing, and only 5.6% had received inhaled corticosteroid (ICS) therapy. During the preceding year, 15.5% of patients experienced at least one emergency department visit and 7.2% required hospitalization because of exacerbation-related respiratory symptoms [3]. These findings indicate substantial gaps in asthma diagnosis, monitoring, and treatment in China and highlight the need for more effective strategies to achieve sustained disease control.

ICS-based therapy remains the foundation of long-term asthma management, while the combination of ICS with a long-acting β_2_-agonist (LABA) is widely recommended as maintenance therapy for patients with moderate-to-severe disease. However, a proportion of patients continue to experience persistent symptoms, impaired lung function, or an increased risk of exacerbations despite conventional ICS/LABA treatment. The 2026 Global Initiative for Asthma (GINA) strategy recommends considering the addition of a long-acting muscarinic antagonist (LAMA) for adults whose asthma remains uncontrolled despite ICS/LABA therapy, although the expected improvements in lung function and exacerbation risk are generally modest [4]. In this context, single-inhaler triple therapy combining an ICS, LABA, and LAMA provides a pharmacologically complementary approach by simultaneously targeting airway inflammation, β_2_-adrenergic-mediated bronchodilation, and cholinergic bronchoconstriction. Fluticasone furoate/umeclidinium/vilanterol (FF/UMEC/VI) is a once-daily single-inhaler triple therapy that integrates the anti-inflammatory activity of fluticasone furoate (FF), the long-acting muscarinic antagonism of umeclidinium (UMEC), and the bronchodilatory effect of vilanterol (VI). FF/UMEC/VI was initially approved in China in 2019 for the maintenance treatment of chronic obstructive pulmonary disease (COPD). In January 2026, the National Medical Products Administration (NMPA) approved its use for the maintenance treatment of asthma in adults, making FF/UMEC/VI the first single-inhaler triple therapy approved in China for both asthma and COPD [5]. This regulatory development has increased the clinical relevance of FF/UMEC/VI for the management of uncontrolled asthma in routine Chinese practice.

The efficacy and safety of FF/UMEC/VI in asthma have been evaluated in the phase IIIA CAPTAIN trial. In patients with uncontrolled moderate-to-severe asthma receiving FF/VI, the addition of UMEC resulted in further improvements in lung function, supporting the clinical value of triple inhaled therapy [6]. However, the CAPTAIN trial enrolled a predominantly non-Asian population, with only 344 of 2436 participants (14%) recruited from Asian regions. Moreover, pharmacokinetic and pharmacodynamic characteristics of inhaled respiratory therapies may vary across ethnic populations, and higher maximum observed plasma concentrations (Cmax) of FF/UMEC/VI have been reported in individuals of East Asian ancestry [7]. These considerations suggest that evidence generated from multinational clinical trials may not fully reflect treatment responses in Chinese patients and support the need for population-specific real-world evaluation.

At present, real-world evidence regarding FF/UMEC/VI in Chinese patients with asthma remains limited. Although real-world data have been reported from Japan and Korea, findings from other East Asian populations may not fully reflect the Chinese clinical setting because of differences in asthma management patterns, allergen sensitization profiles, and treatment accessibility. FF/UMEC/VI was approved for asthma in China only a few months ago, and real-world data on its use in Chinese patients with asthma are currently lacking. Therefore, this retrospective real-world study primarily described changes in lung function and safety following FF/UMEC/VI (100/62.5/25 μg) in Chinese patients with asthma requiring treatment intensification at a tertiary allergy center. By providing real-world evidence from a Chinese clinical setting, this study may provide an initial basis for the clinical application of FF/UMEC/VI in China and help generate hypotheses for future prospective studies.

## 2. Materials and Methods

This retrospective, single-center, real-world study with a before-after design evaluated the effectiveness and safety of FF/UMEC/VI in Chinese patients with uncontrolled asthma. The study population, outcome measures, and statistical methods are described below.

### 2.1. Patients

This study was carried out at Peking Union Medical College Hospital (PUMCH) in China from July 2021 through March 2024. Eligible participants were asthma patients seen at the Allergy outpatient clinic who had initiated treatment with FF/UMEC/VI (100/62.5/25 μg) and for whom at least one evaluable assessment was available both prior to and following the start of therapy. This is a retrospective, observational study using de-identified medical records. Clinical and demographic data were extracted from electronic medical records. The study does not involve direct patient contact, does not infringe on personal privacy, and carries no commercial interests. In accordance with the Guidelines for the Construction of Ethics Review Committees for Human Clinical Research (2020 Edition), our IRB granted a full waiver of informed consent because: the risk to subjects is no more than minimal and waiving consent does not adversely affect subjects’ rights and welfare. The protocol adhered to the ethical standards set forth in the Declaration of Helsinki, received approval from the PUMCH Institutional Review Board (approval reference: K24C1461, approval date: 6 May 2024).

### 2.2. Outcome Measures

Demographic and clinical characteristics, previous medication use, allergy status, pulmonary function, adverse events, and subsequent treatment changes were recorded. The primary outcome was the change in forced expiratory volume in 1 s (FEV_1_) from baseline to 1 month after treatment initiation. Secondary outcomes included changes in FEV_1_, percent predicted FEV_1_, and the FEV_1_/forced vital capacity (FEV_1_/FVC) ratio at subsequent follow-up visits and safety. Particular attention was paid to changes in FEV_1_ and FEV_1_/FVC before and after FF/UMEC/VI initiation. The proportions of patients achieving a percent predicted FEV_1_ ≥ 70% or an FEV_1_/FVC ≥ 0.70 after treatment initiation were also evaluated. All spirometry assessments were performed more than 24 h after the last dose of FF/UMEC/VI.

### 2.3. Statistical Analysis

Descriptive statistics for continuous variables are expressed as means ± standard deviations (SDs). Normality of the data was evaluated using the Shapiro–Wilk test, and equality of variances was checked before any parametric analysis was performed. The primary longitudinal analysis used a linear mixed-effects model for each spirometric outcome. All available post-baseline observations were included, and missing data were not imputed. Complete-case repeated-measures ANOVA with Tukey’s post hoc procedure was used as a sensitivity analysis when assumptions were met. In cases where parametric criteria were not met, non-parametric alternatives—including the Mann–Whitney U test, Friedman test, or Kruskal–Wallis test—were adopted, and Dunn’s multiple-comparison test was used as appropriate. A two-sided *p*-value < 0.05 was defined as statistically significant. All statistical computations were carried out using GraphPad Prism 9.0 (GraphPad Software, Boston, MA, USA) and SPSS 24.0 (IBM, Armonk, NY, USA).

## 3. Results

Thirty patients with asthma were included in the final analysis. The clinical characteristics and outcomes following FF/UMEC/VI treatment are summarized in the following sections.

### 3.1. Baseline Characteristics

A total of 30 patients with asthma were included in the analysis. The mean age was 40.53 ± 15.74 years (range, 15–76 years), and 15 patients (50%) were female. All participants were of East Asian ancestry. Twenty-eight patients (93.3%) had asthma alone, whereas 2 (6.7%) had asthma–COPD overlap. Serum total IgE levels ranged from 2.7 to >5000 kU/L. Twenty-five patients (83.3%) had total IgE levels ≥ 60 kU/L. Aeroallergen sensitization was identified in 26 patients (86.7%), including 10 (33.3%) with sensitization to a single allergen and 16 (66.7%) with sensitization to multiple allergens. The most common sensitizing allergens were house dust mites (18 patients, 60%), followed by animal dander (12, 40%), fungi (7, 23.3%), pollen (7, 23.3%), and cockroach (3, 10%) (Table 1).

**Figure 1:**
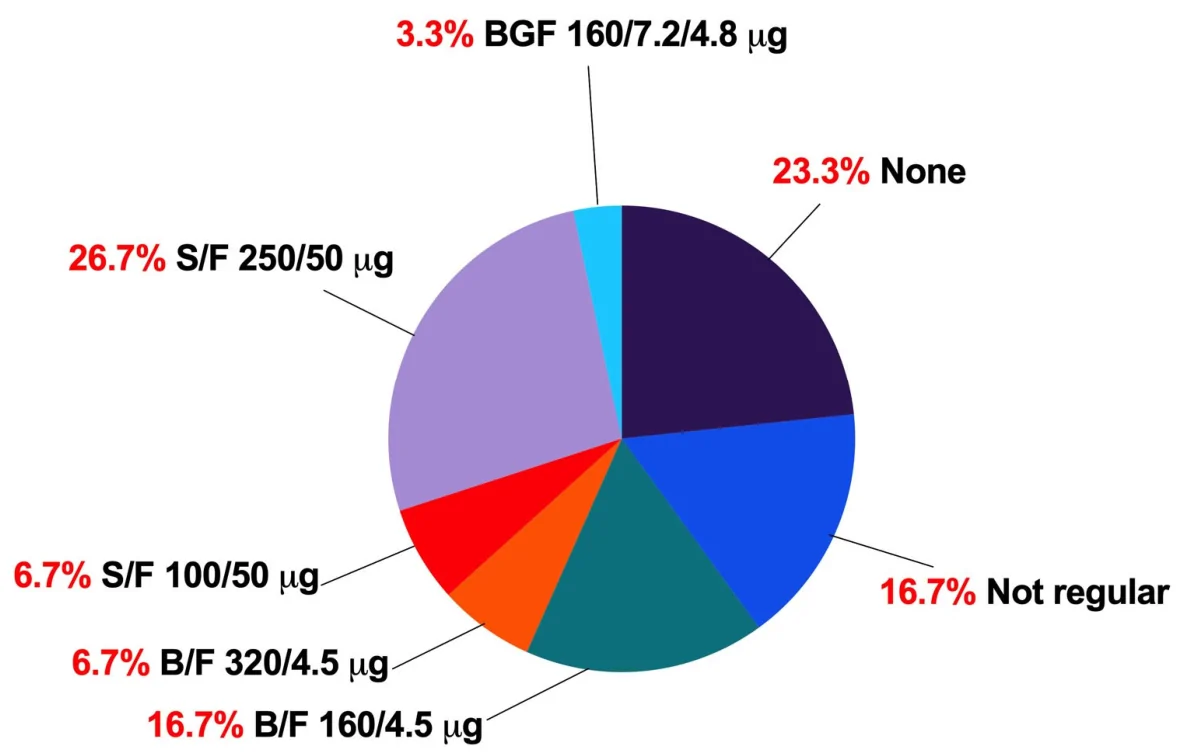
**Prior Inhalation Medications Before Single-FF/UMEC/VI Initiation (n = 30).** Distribution of inhaled controller therapies used before the initiation of single-inhaler FF/UMEC/VI 100/62.5/25 μg. Seven (23.3%) subjects with asthma had not used inhaled drugs. Five (16.7%) subjects with asthma used inhalation drugs not regularly. B/F, budesonide with formoterol fumarate; BGF, budesonide with glycopyrrolate and formoterol fumarate; FF/UMEC/VI, fluticasone furoate/umeclidinium/vilanterol; and S/F, salmeterol xinafoate with fluticasone propionate.

Before FF/UMEC/VI initiation, 7 patients (23.3%) had not used inhaled medication, whereas 5 (16.7%) reported irregular use. Among patients receiving regular inhaled therapy, the most frequently used regimen was salmeterol/fluticasone propionate (S/F) 250/50 μg (26.7%), followed by budesonide/formoterol fumarate (B/F) 160/4.5 μg (16.7%), B/F 320/4.5 μg (6.7%), S/F 100/50 μg (6.7%), and budesonide/glycopyrrolate/formoterol fumarate (BGF) 160/7.2/4.8 μg (3.3%) (Figure 1).

### 3.2. Lung Function Changes

**Figure 2:**
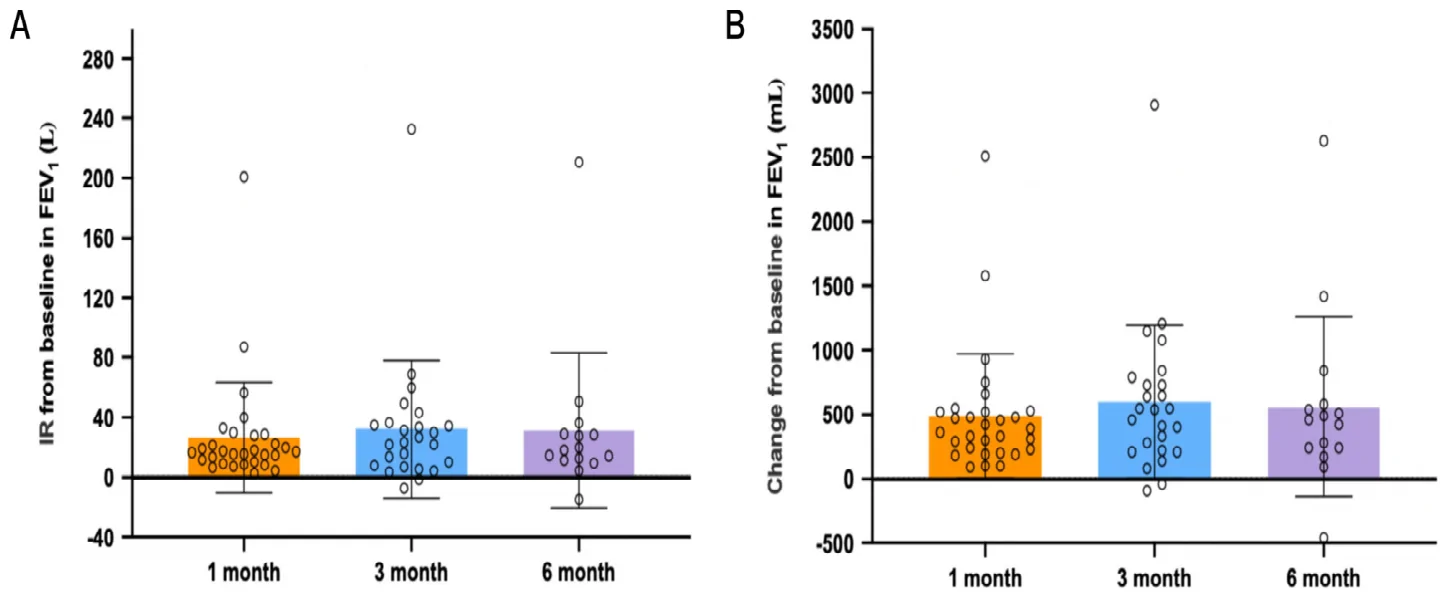
**Changes in FEV_1_ Following Treatment with Single-Inhaler FF/UMEC/VI.** (**A**) Improvement rate in FEV_1_ and (**B**) mean change in FEV_1_ from baseline at 1, 3, and 6 months after treatment initiation. FF/UMEC/VI, fluticasone furoate/umeclidinium/vilanterol. Data are shown as mean ± SD **(30 at baseline, 30 at 1 month, 25 at 3 months, and 15 at 6 months for spirometry analysis).**

**Figure 3:**
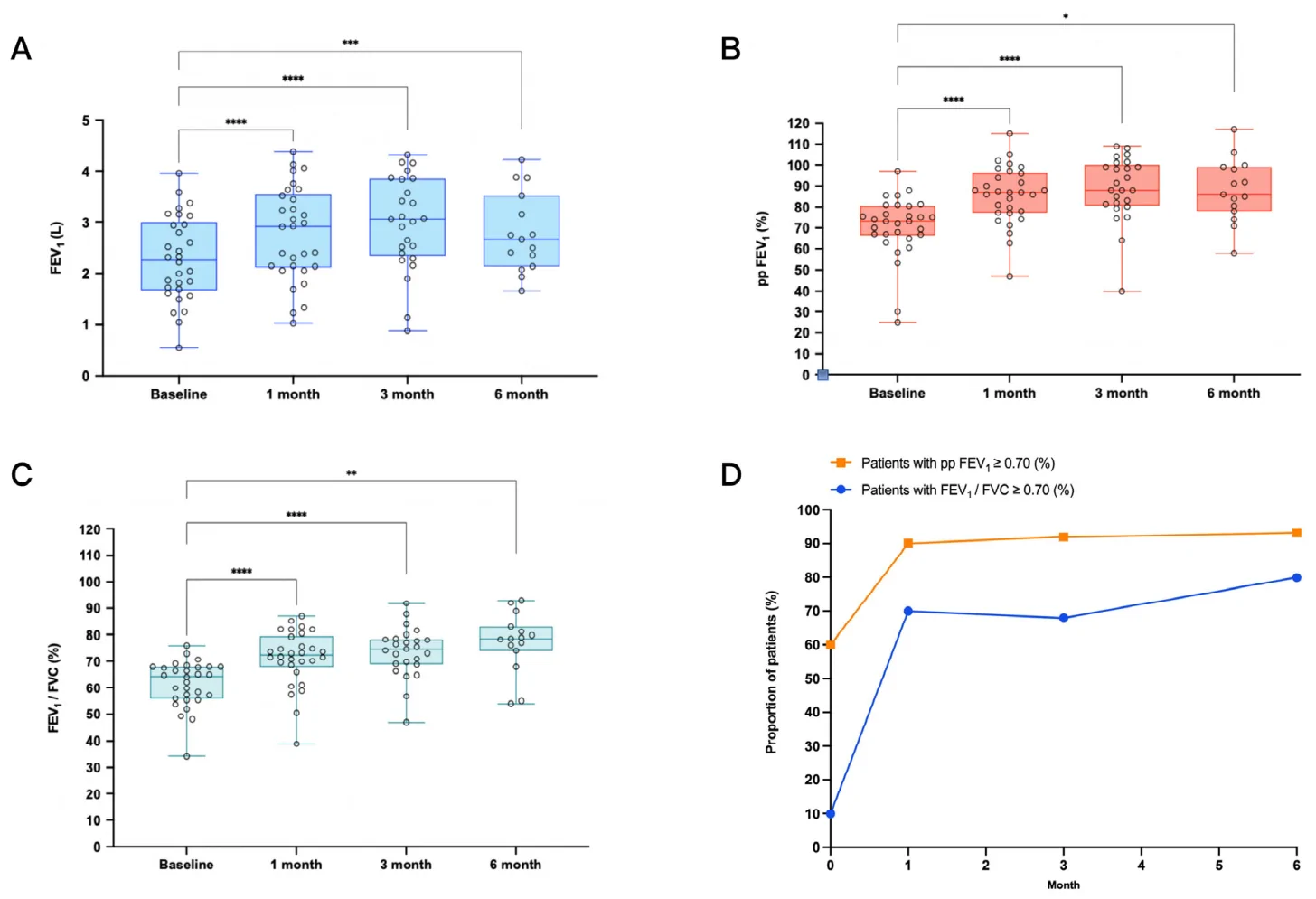
**Changes in Lung Function Parameters and Responder Proportion after the Use of Single-FF/UMEC/VI.** (**A**) FEV_1_ (L), (**B**) pp FEV_1_ (%), and (**C**) FEV_1_/FVC (%) before and after 1, 3, and 6 months of treatment with single-FF/UMEC/VI. (**D**) Proportion of patients reaching pp FEV1 ≥ 0.70 (%) or FEV1/FVC ≥ 0.70 (%) after using single-FF/UMEC/VI for 1, 3, and 6 months. FEV1, percent predicted forced expiratory volume in 1 s; ppFEV1, percent predicted FEV1; FVC, forced vital capacity. Data are shown as mean ± SD. * *p* < 0.05, ** *p* < 0.01, *** *p* < 0.001, **** *p* < 0.0001 **(30 at baseline, 30 at 1 month, 25 at 3 months, and 15 at 6 months for spirometry analysis)**.

Baseline mean FEV1 was 2.29 ± 0.83 L (range, 0.55–3.96 L). Compared with baseline, FEV_1_ increased by 16.88%, 23.32%, and 18.03% at 1, 3, and 6 months, respectively (Figure 2A). The corresponding absolute increases in mean FEV_1_ were 375, 540, and 460 mL (Figure 2B). FEV_1_ improved significantly after 1 month of FF/UMEC/VI treatment and remained significantly higher than baseline at 3 and 6 months (*p* < 0.0001, *p* < 0.0001, and *p* < 0.001, respectively) (Figure 3A). Percent predicted FEV_1_ also increased significantly at 1, 3, and 6 months (*p* < 0.0001, *p* < 0.0001, and *p* = 0.036, respectively) (Figure 3B). Similarly, FEV_1_/FVC improved significantly at each follow-up time point (*p* < 0.0001, *p* < 0.0001, and *p* = 0.008, respectively) (Figure 3C). At baseline, 60% of patients had a percent predicted FEV_1_ ≥ 70%. This proportion increased to 90%, 92%, and 92.3% at 1, 3, and 6 months, respectively. The proportion of patients with FEV_1_/FVC ≥ 0.70 increased from 10% at baseline to 70%, 68%, and 80% at 1, 3, and 6 months, respectively (Figure 3D). In the two patients with asthma-COPD overlap, FEV_1_ improved by 87.27% and 13.33% from baseline at 1 month, respectively. Given the very small number, no formal subgroup analysis was performed (30 at baseline, 30 at 1 month, 25 at 3 months, and 15 at 6 months for spirometry analysis).

### 3.3. Safety

Six patients (20%) were lost to follow-up because they did not return for follow-up visits and could not be reached by telephone. No serious adverse events were recorded during the available follow-up period. Seven patients (23.3%) reported throat discomfort and sputum-related symptoms. These events occurred within the first 4 weeks of treatment, were mild in severity, and resolved spontaneously or with symptomatic measures. None required treatment discontinuation. None of these events were considered related to loss to follow-up. However, the small sample size and relatively short follow-up limit the ability to detect uncommon or delayed adverse events.

## 4. Discussion

### 4.1. Rapid Improvement After FF/UMEC/VI

In this single-center real-world study, treatment with FF/UMEC/VI was associated with significant improvements in FEV_1_, percent predicted FEV_1_, and FEV_1_/FVC in Chinese patients with uncontrolled asthma. These improvements were evident within 1 month and were maintained throughout the available 6-month follow-up, broadly supporting the pulmonary function benefits reported in previous clinical studies [8,9,10]. The findings are also consistent with the CAPTAIN trial, in which the addition of UMEC to FF/VI improved lung function in patients with inadequately controlled moderate-to-severe asthma [6]. However, CAPTAIN did not demonstrate a significant reduction in moderate or severe exacerbations with the addition of UMEC, whereas the effect of higher-dose FF on exacerbation outcomes appeared more relevant in selected inflammatory phenotypes. Therefore, the present findings should primarily be interpreted as supporting the real-world effectiveness of FF/UMEC/VI in improving pulmonary function rather than as evidence of reduced exacerbation risk. In patients with no prior inhaled medication and irregular inhaled therapy, the observed improvement may represent rapid asthma control after initiation of FF/UMEC/VI. In patients with regular inhaled therapy but inadequate control, improvement may more plausibly reflect the stronger therapeutic effect of FF/UMEC/VI after conventional inhaled treatment failed. However, because of the small sample size and lack of adjustment for confounders, these subgroup interpretations are descriptive and hypothesis-generating.

A potential practical benefit of single-inhaler triple therapy is that ICS, LABA, and LAMA can be administered through one device, which may simplify treatment and reduce the complexity associated with multiple inhalers [11]. Compared with biologic therapies, inhaled triple therapy may also be more readily implemented in routine clinical practice. However, our study did not include a formal cost-effectiveness assessment, and therefore no quantitative conclusions regarding economic advantages can be drawn. Current evidence supports single-inhaler triple therapy as a treatment-intensification option for patients who remain uncontrolled despite ICS/LABA therapy, provided that adherence, inhaler technique, comorbidities, and treatment intensity have been appropriately reassessed. Consistent with the 2026 GINA strategy, the benefits of adding a LAMA are generally more evident for lung function than for symptom improvement, emphasizing the importance of optimizing existing therapy before treatment escalation [4]. In real-world practice, clinicians are often hesitant to initiate triple therapy due to concerns about overtreatment, potential side effects, and cost. Actually, triple therapy can be used as an initial intensification strategy, but regular assessment and adjustment of therapy are necessary to maintain control with the minimum effective dose. Prospective studies are needed to establish the optimal timing and criteria for reducing treatment intensity.

### 4.2. Safety and Tolerability in Real-World Practice

FF/UMEC/VI was generally well tolerated in this real-world cohort. No serious adverse events were recorded during the available follow-up, while throat discomfort and sputum-related symptoms were the main reported adverse events [12]. These findings are broadly consistent with the established safety profile of inhaled triple therapy. Nevertheless, the small sample size and follow-up duration are insufficient to draw definitive conclusions about long-term safety. Larger studies with longer observation periods are therefore required to provide a more comprehensive assessment of the long-term safety of FF/UMEC/VI in Chinese patients with asthma.

### 4.3. Limitations

Several methodological caveats merit consideration when interpreting the present results. First, the retrospective, single-center design and relatively modest sample size render the study susceptible to selection and information biases and also limit the broader applicability of our observations. Baseline asthma characterization was incomplete, including ACT (Asthma Control Test)/ACQ (Asthma Control Questionnaire) scores and symptom burden. Fourth, adherence and inhaler technique were not systematically assessed. The cohort was heterogeneous with respect to age, prior inhaled therapy, and asthma phenotype, but the small cohort further precluded any robust subgroup exploration. Second, in the absence of a concurrent control arm, it is difficult to ascribe the observed improvements solely to FF/UMEC/VI, since concomitant factors—such as better medication adherence, improved inhalation technique, or adjunctive therapies—may have confounded the outcomes. Third, the follow-up duration was brief, and the protocol was not intended to capture long-term endpoints like exacerbation rates or sustained dose reduction. Fourth, longitudinal spirometry data were partially missing; therefore, available-case analyses may be biased, and the 6-month findings should be considered exploratory. In addition, a 20% loss to follow-up raises the possibility of attrition bias, and we could not determine whether these patients continued, changed, or discontinued treatment, or whether they experienced adverse events. Lastly, the diversity of prior inhaled corticosteroid-containing regimens across participants might have contributed to interindividual variability in therapeutic response.

### 4.4. Future Directions

Future studies should investigate whether baseline clinical characteristics, inflammatory phenotype, allergy status, small-airway function, and exacerbation history can identify patients most likely to benefit from FF/UMEC/VI. Multicenter prospective studies with larger cohorts and longer follow-up are warranted to validate the observed improvements in pulmonary function and to determine whether treatment intensification can be followed safely by step-down in appropriately selected patients. Comparative studies involving other triple inhaled therapies and biologics in well-defined patient subgroups may further clarify the appropriate positioning of FF/UMEC/VI in the asthma treatment pathway. In addition, health-economic studies in the Chinese healthcare setting would help determine its potential value from a cost-effectiveness perspective.

## 5. Conclusions

In Chinese patients with uncontrolled asthma, real-world treatment with single-inhaler FF/UMEC/VI was associated with improved lung function and no serious adverse events were identified in this cohort. Larger prospective multicenter studies are required to validate these findings, clarify long-term outcomes, and identify the optimal patient population for this treatment.

## Figures and Tables

**Table 1:** Baseline Characteristics of the 30 Asthma Patients.

Epidemiological Characteristics	
Age, mean ± SD (range), years	40.53 ± 15.74 (15~76)
<18 years, n (%)	2 (6.7%)
≥18–<65 years, n (%)	26 (86.7%)
≥65 years, n (%)	2 (6.7%)
Female, n (%)	15 (50%)
Race, n (%)	
East Asian heritage, n (%)	30 (100%)
Diagnose	
Asthma, n (%)	28 (93.3%)
Asthma-COPD overlap, n (%)	2 (6.7%)
Course of asthma, medium (interquartile range), years	8 (4~10)
Exacerbations requiring oral corticosteroids, n (%)	7 (23.3%)
Exacerbations requiring hospitalization, n (%)	5 (16.7%)
Comorbidities	
Allergic rhinitis, n (%)	24 (80%)
Chronic rhinosinusitis, n (%)	6 (20%)
Serum total IgE	
Range (kU/L)	2.7~2313, one patient > 5000
<60 kU/L, n (%)	5 (16.7%)
≥60 kU/L, n (%)	25 (83.3%)
Aeroallergens	
Sensitization to aeroallergens, n (%)	26 (86.7%)
Single-allergen hypersensitivity, n (%)	10 (33.3%)
Multi-allergen hypersensitivity, n (%)	16 (66.7%)
House dust mites, n (%)	18 (60%)
Animal dander, n (%)	12 (40%)
Fungus, n (%)	7 (23.3%)
Pollen, n (%)	7 (23.3%)
Cockroach, n (%)	3 (10%)

## Data Availability

The data that support the findings of this study are available from the corresponding author upon reasonable request.

## Abbreviations

The following abbreviations are used in this manuscript:

ICS	Inhaled corticosteroid
LABA	long-acting β_2_-agonist
FF/UMEC/VI	fluticasone furoate/umeclidinium/vilanterol
FEV_1_	forced expiratory volume in 1 s
FVC	forced vital capacity
GINA	Global Initiative for Asthma
LAMA	long-acting muscarinic antagonist
COPD	chronic obstructive pulmonary disease
NMPA	National Medical Products Administration
Cmax	maximum observed concentration
S/F	salmeterol/fluticasone propionate
B/F	budesonide/formoterol fumarate
BGF	budesonide/glycopyrrolate/formoterol fumarate
